# Prognostic Value of Two Polymorphisms, *rs1045642* and *rs1128503*, in *ABCB1* Following Taxane-based Chemotherapy: A Meta-Analysis

**DOI:** 10.31557/APJCP.2021.22.1.3

**Published:** 2021-01

**Authors:** Quanyao Chen, Wanlong Lin, Jianhui Yang, Min Lin, Xiuxian Lin, Yiyin Weng, Yao Chen

**Affiliations:** *Women and Children’s Hospital, School of Medicine, Xiamen University, Xiamen, Fujian, China.*

**Keywords:** Paclitaxel, taxane, ABCB1, progression, free survival, overall survival

## Abstract

**Objective:**

Genetic polymorphisms can influence the chemotherapeutic response; however, previous studies have produced conflicting results, and have failed to identify the most relevant polymorphisms for predicting the response to treatment in patients with cancer. The present meta-analysis was conducted to determine the correlation between two polymorphisms (*rs1045642* and *rs1128503*) in ATP-binding cassette transporter B subfamily member 1 (*ABCB1*), which is associated with multidrug resistance, and the survival of patients treated with taxane-containing chemotherapy.

**Methods:**

Several databases, including PubMed and Embase, were used to retrieve articles evaluating the association between the *ABCB1 rs1045642 *and *rs1128503* polymorphisms and survival, published prior to August 2019. The meta-analysis was conducted using R software to determine the pooled hazard ratio (HR) and 95% confidence intervals (95% CIs).

**Results:**

Fifteen studies involving 3320 patients were included in the meta-analysis. The effect of the *rs1128503* polymorphism on progression-free survival remained significant in the heterozygote (HR 0.81; 95% CI: 0.67–0.98) and homozygote (HR 0.71; 95% CI: 0.58–0.88) models. The TT genotype *rs1128503 *was associated with better overall survival (HR 0.72; 95% CI: 0.53–0.97).

**Conclusion:**

Carriers of the rs1128503 T allele of *ABCB1 *showed a survival benefit after taxane-containing chemotherapy.

## Introduction

Taxanes, a class of diterpenes, are widely used to treat different types of solid tumors, such as breast cancer, ovarian cancer, some head and neck cancers, and lung cancer. Taxanes exert their anti-tumor effects by disrupting the dynamic balance between microtubules and tubulin dimers, promoting tumor cell apoptosis. Clinical studies have shown that the toxicity and efficacy of taxanes vary widely and exhibit inter-individual differences, leading to substantial variations in individual prognosis. An increasing number of studies have indicated that genetic factors such as single nucleotide polymorphisms (SNPs) play important roles in explaining the individual differences in the prognosis of patients administered taxane-containing chemotherapy (Johnatty et al., 2013; Karageorgopoulou et al., 2017; Szczyrek et al., 2017; Bjorn and Jakobson Falk, 2018).

Several genetic polymorphisms have been shown to affect the activity of taxanes. The most common gene reportedly associated with these effects is ATP-binding cassette transporter B subfamily member 1 (*ABCB1*), also known as multidrug resistance gene 1, which is located on the long arm of human chromosome 7. *ABCB1* encodes P-glycoprotein (P-gp), which can transport a variety of drugs, and also functions as a transmembrane osmotic pump (Leschziner et al., 2007). Upon an increase in P-gp activity, antitumor drugs that enter the cell can be actively pumped out of the cell (Dean et al., 2001; Kelland, 2007; Auner et al., 2010), leading to increased outflow and/or decreased inflow, so that the antitumor drugs do not accumulate at sufficiently high levels in tumor cells, thereby reducing their efficacy; this is the key mechanism by which P-gp exerts chemotherapy resistance (Kim et al., 2001; Genovese et al., 2017). P-gp is involved in the transport of multiple antitumor drugs, including paclitaxel and docetaxel (Orina et al., 2009). Therefore, SNPs in ABCB1 may affect the function of P-gp to ultimately influence drug sensitivity and drug resistance, through mechanisms (Liu et al., 2013; Xia et al., 2016) such as alteration of the expression levels, stability, degradation, substrate specificity, activity, and/or role of transporters (Hoffmeyer et al., 2000; Hitzl et al., 2001; De Iudicibus et al., 2008; Schaich et al., 2009), with consequent impacts on prognosis (Mizuno et al., 2003). 

The association between *ABCB1* polymorphisms and the prognosis of patients following taxane-containing chemotherapy has been explored in several studies. However, the findings are inconclusive, with contrasting results and weak data (Grau et al., 2009; Bergmann et al., 2011; Qiao et al., 2016; Li et al., 2017; Priyadarshini et al., 2019; Zhong et al., 2019). Given the potential importance of *ABCB1 *polymorphisms in the response to chemotherapy, we conducted a meta-analysis of studies conducted to date, along with data obtained by doctors, pharmacists, and statisticians, to systematically integrate the current pharmacogenetic literature and obtain more credible evidence. In particular, we focused on the association between the *ABCB1 SNPs rs1045642 *(*C3435T*) and *rs1128503* (*C1236T*) and the prognosis of patients administered taxane-based chemotherapy, to provide a basis for personalized clinical medicine.

## Materials and Methods

*Data sources, search strategy, and selection criteria*

This study was performed according to the Preferred Reporting Items for Systematic Reviews and Meta-Analyses (PRISMA) the Meta-analysis of Observational Studies in Epidemiology guidelines (Moher et al., 2009) (PROSPERO Registration Number: CRD42019128195). The electronic databases PubMed, Embase, Web of Science, Cochrane library, Chinese National Knowledge Infrastructure, Wanfang, and Weipu were searched systematically from the time of library construction to August 2019 by two authors. The basic search was restricted to English-language articles with the following core search terms: (taxane or taxol or paclitaxel or docetaxel or albumin-bound or protein-bound) and (outcomes or “overall survival” or OS or “progression-free survival” or PFS) and (“multidrug resistance gene” or ABCB1 or MDR1). To identify additional published studies and unpublished literature, we manually searched the reference lists of the retrieved studies or relevant reviews, and the authors were contacted. Only the most recent study was included when overlap occurred between studies.

Studies were considered to be eligible if they fulfilled all of the following criteria: all included patients were diagnosed with a solid tumor; all included patients were administered taxane-based chemotherapy; the studies included comparisons of different categories of the polymorphic genotypes *ABCB1 C3435T* and *C1236T*; all reported the overall survival (OS) and progression-free survival (PFS) of patients with different genotypes, and had sufficient genotype data to estimate the hazard ratio (HR) and 95% confidence interval (CI) in at least one genetic comparison model. The study selection process was performed by two authors independently; a third author determined the final criteria for any inconsistencies.

*Data collection and quality assessment*

Two authors were responsible for extracting the data from eligible studies using a standardized data extraction table. Disagreements were resolved by group discussion, or by a third author if a consensus could not be reached. Information on the first author’s name, publication year, country, patient mean age, sample size, tumor type, regimen, detection, Hardy-Weinberg equilibrium, PFS, and OS for each category of genotypes were collected. Bias caused by individual studies was examined by two authors independently using the Newcastle–Ottawa Scale score, which is useful for comprehensively evaluating the quality of observational studies in a meta-analysis. The bias of selection (four items), comparability (one item), and outcome (three items) were assessed during this process.

*Data synthesis and analysis*

Three genetic models were analyzed for each SNP in this meta-analysis: a homozygote model (TT vs. CC), a heterozygote model (CT vs. CC), and a dominant model (CT + TT vs. CC). For time-to-event survival analysis, we assessed the effect of ABCB1 status on patient prognosis by calculating the HR. For each study, the HR and its 95% CI were retrieved. If these parameters were not available in the articles, we used the software Engauge Digitizer 4.1 to extract specific survival rates according to Kaplan–Meier curves and calculate the HR, as described by Tierney et al., (2007). Heterogeneity in the pooled analyses was determined via statistical analyses, using the Q statistic for homogeneity and the I^2^ statistic. A P value of < 0.10 or I^2^ > 50% was considered to indicate significant heterogeneity (Higgins et al., 2003). When statistical heterogeneity existed, the random-effects model was used; otherwise, the fixed-effects model was applied. Sensitivity analyses were performed to evaluate the influence of single studies on the overall analysis. Subgroup analyses, including country (Asia, Europe, and others), diagnosis (ovary, breast, lung, and others), and regimen [paclitaxel plus cisplatin (TP) and others] were conducted. Publication biases were estimated using Egger tests (Egger et al., 1997). A two-sided P value of less than 0.05 was taken to indicate statistical significance. All statistical analyses were performed using R version 3.6.1 software.

## Results

*Search results*

**Figure 1:**
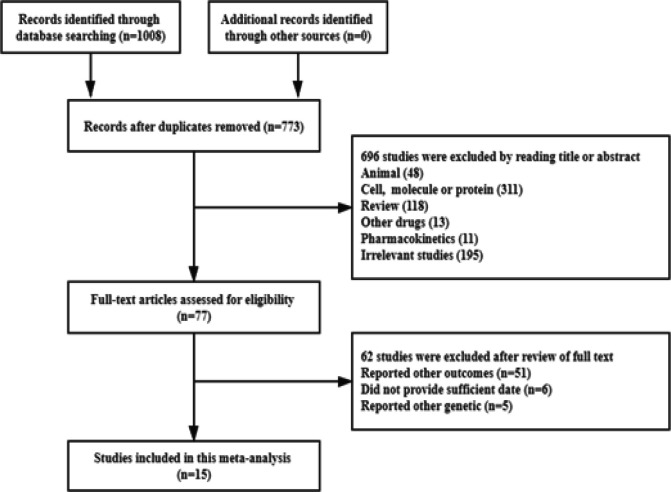
Flow Diagram of the Literature Search and Trial Selection Process

Figure 1 presents the entire process of study selection. A total of 1,008 potentially relevant articles were identified by systematic searching of electronic databases, and manual searching. After reviewing the titles or abstracts, 235 studies were excluded as duplicates, leaving 773 articles for further title and abstract review. A total of 77 studies were discarded following a review of the full text. Fifteen studies were finally identified and included in the analysis. The remaining studies were excluded because of incompatible data, nonrelevant genotypes, and focus on other outcomes (Johnatty et al., 2008; Chang et al., 2009; Gandara et al., 2009; Grau et al., 2009; Chang et al., 2010; Shim et al., 2010; Bergmann et al., 2011; Peethambaram et al., 2011; Kim et al., 2012; Tiam et al., 2012; Johnatty et al., 2013; Qiao et al., 2016; Li et al., 2017; Szczyrek et al., 2017; Bjorn and Jakobsen Falk, 2018).

*Study characteristics*

The characteristics of all studies included are listed in Table 1. Fifteen studies reporting data from 3320 patients who were administered taxane-containing chemotherapy were included in this study. The years of publication ranged from 2008 to 2018. The sample sizes ranged from 43 to 511 patients, and their mean ages ranged from 46 to 65 years. Table 1 also provides information on the country, sample size, diagnosis, tumor type, regimen used, detection, and Newcastle–Ottawa Scale score. Study quality was assessed using the Newcastle–Ottawa Scale score: one study had a score of 6, 11 studies had a score of 8, and the remaining five studies had a score of 9.

*ABCB1 C3435T polymorphism*

*PFS*

**Figure 2:**
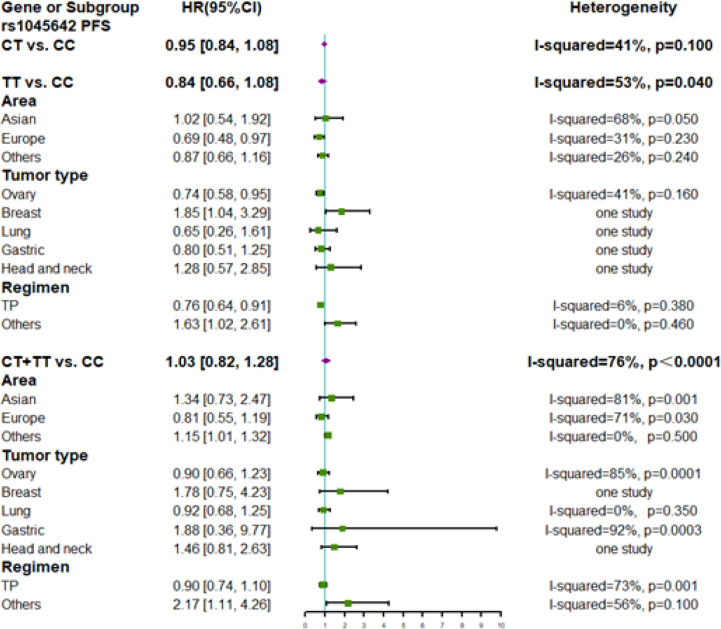
Forest Plots of ABCB1 rs1045642 Polymorphism and Subgroup Analyses on Progression-Free Survival of Patients Administered Taxane-Based Chemotherapy

Eleven studies involving 2670 patients were included in the meta-analysis for PFS (Figure 2). The C3435T polymorphism showed no correlation with PFS. Subgroup analysis was performed to evaluate different countries, tumor types, and regimens. The CC genotype had a predictive effect on OS (HR 0.69; 95% CI: 0.48–0.97) in Europe compared to TT carriers. Patients with ovarian cancer who were CC carriers showed poor PFS (HR 0.74; 95% CI: 0.58–0.95) compared to that of TT carriers. Patients who were CC carriers showed poor PFS (HR 0.76; 95% CI: 0.64–0.91) following TP regimen-based chemotherapy compared to that of CT carriers, and better PFS (HR 1.63; 95% CI: 1.02–2.61) following TP regimen-based chemotherapy compared to that of TT carriers. Patients who were CT + TT carriers showed a poor PFS (HR 2.17; 95% CI: 1.11–4.26) following non-TP regimen chemotherapy compared to that of CC carriers. Significant heterogeneity was found in the overall meta-analysis of the homozygote model (I^2^ = 53%, P = 0.04) and dominant model (I^2^ = 76%, P = 0.04). Sensitivity analyses showed that heterogeneity was decreased in the heterozygous model (I^2^ = 12%, P = 0.34) after excluding the study by Li et al., (2017).

*OS*

**Figure 3:**
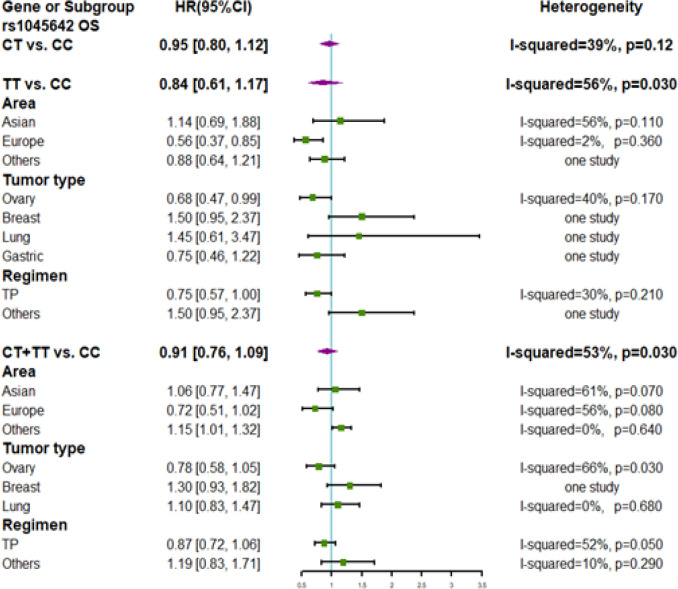
Forest Plots of ABCB1 rs1045642 Polymorphism and Subgroup Analyses for Overall Survival of Patients Administered Taxane-Based Chemotherapy

Nine studies involving 2255 patients were included in the meta-analysis for OS (Figure 3). The *C3435T* polymorphism was not associated with an improvement in OS among patients treated with taxane-containing chemotherapy. Results of the subgroup analysis showed that European patients who were CC carriers had a poor OS (HR 0.56; 95% CI: 0.37–0.85) compared to that of TT carriers. Patients with ovarian cancer who were CC carriers showed a poor OS (HR 0.68; 95% CI: 0.47–0.99) compared to that of TT carriers. Significant heterogeneities were found in the overall meta-analysis of the homozygote model (I^2^ = 56%, P = 0.03) and dominant model (I^2^ = 53%, P = 0.03). Sensitivity analyses showed that heterogeneity was decreased in the heterozygous model (I^2^ = 30%, P = 0.21) after excluding the study by Li et al. (2017) and was decreased in the dominant model (I^2^ = 7%, P = 0.0.37) after excluding the study by Bjorn et al., (2018) (Arm A). 

*ABCB1 C1236T polymorphism*

*PFS*

**Figure 4:**
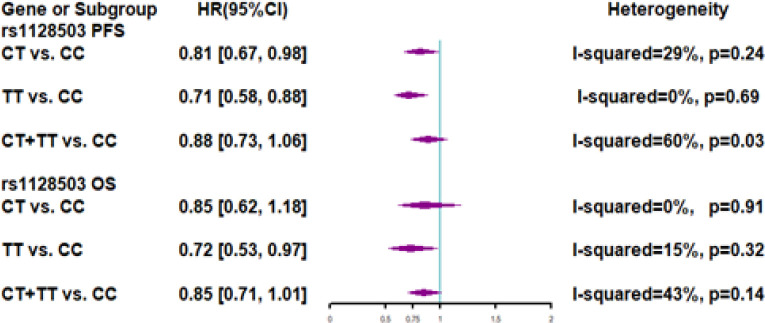
Forest Plots of ABCB1 rs1128503 Polymorphism and Subgroup Analyses for Progression-Free Survival and Overall Survival of Patients Administered Taxane-Based Chemotherapy

Seven studies involving 1943 patients were included in the meta-analysis for PFS (Figure 4). The summary results showed that the effect of the C1236T polymorphism on PFS remained significant in the heterozygote model (HR 0.81; 95% CI: 0.67–0.98) and homozygote model (HR 0.71; 95% CI: 0.58–0.88). Significant heterogeneity was found in the dominant model (I^2^ = 60%, P = 0.03). Sensitivity analyses showed that heterogeneity was decreased in the dominant model (I^2^ = 41%, P = 0.15) after excluding the study by Johnatty et al., (2013).

*OS*

Six studies involving 1,217 patients were included in the meta-analysis for OS (Figure 4). The CC genotype showed a predictive effect on OS. Compared to the TT phenotype, the CC genotype was associated with a poor OS (HR 0.72; 95% CI: 0.53–0.97). There was significant heterogeneity identified among studies.

*Publication bias*

No significant publication bias was observed. 

**Table1:** Baseline Characteristics of the Studies Included in the Meta-Analysis

Study	Country	Size M/F(n) Age	Tumor type	Regimen (Dose)	Detection	HWE	End-point	Gene	NOS
Bergamnn 2011	Denmark/ Sweden	119 N/A 57 (26–77)	Ovary	TP (175 mg/m^2^ and AUC5-6)	Pyrosequencing	P >0.05	OS	C3435T	8
Bjorn (Arm A) 2018	Sweden	260 N/A 56 (26-81)	Ovary	TP (250 mg/m^2^ and AUC5-6)	Pyrosequencing	P >0.05	PFS/OS	C3435T C1236T	8
Bjorn (Arm B) 2018	Sweden	265 N/A 56 (37-81)	Ovary	TP (175 mg/m^2^ and AUC5-6)	Pyrosequencing	P >0.05	PFS/OS	C3435T C1236T	8
Chang 2009	Korea	108 N/A 49 (32–71)	Breast	Paclitaxel (175 mg/m^2^)	Sanger	P >0.05	OS	C3435T	8
Chang 2010	Korea	43 26/17 47 (23-68)	Gastric	Paclitaxel (175 mg/m^2^) + Leucovorin (20 mg/m^2^) + 5-fluorouracil (1000 mg/m^2^)	Sanger	P <0.05	PFS	C3435T	6
Gandara 2009	Japan	197 136/61 65 (33–81)	Lung	TP (225 mg/m^2^ and AUC6)	Pyrosequencing	P >0.05	PFS/OS	C3435T	8
	American	184 116/68 63 (28–80)							
Grau 2009	Spain	47 43/4 57 (46–80)	Head and neck	Paclitaxel (80 mg/m^2^)	Primers and probes	P >0.05	PFS	C3435T C1236T	8
Johnatty 2008	Austria	309 N/A 58(31-80)	Ovary	TP (175 or 135 mg/m^2^ and AUC5-6)	MALDI-TOF	P >0.05	PFS/OS	C3435T C1236T	9
Johnatty 2013	Austria	433 N/A N/A	Ovary	TP (175 or 135 mg/m2 and AUC5-6)	Illumina Inﬁnium iSelect array	P >0.05	PFS	C3435T C1236T	9
Kim 2012	Korea	57 N/A 46 (27–72)	Breast	Paclitaxel +trastuzumab (80 mg/m^2^ + load 4 mg/kg maintain 2 mg/kg weekly; 175 mg/m^2^ + load 8 mg/kg maintain 6 mg/kg 3 weeks) Docetaxel + trastuzumab 75 mg/m^2^ + load 8 mg/kg maintain 6 mg/kg 3 weeks)	PCR-RFLP	P >0.05	PFS/OS	C3435T	8
Li 2017	Chinese	100 N/A 50 (23-77)	Breast	Docetaxel and Epirubicin (NA)	TaqMan	P >0.05	PFS/OS	C3435T	9
Prema 2011	American	365 N/A N/A	Ovary	Paclitaxel or Docetaxel (NA)	Veracode Assay	P >0.05	PFS	C1236T	9
Qiao 2016	Chinese	64 44/20 58 (38-73)	Lung	TP (175 mg/m^2^ and AUC5-6)	Sequenom iPLEX Mass ARRAY Platform	P >0.05	PFS/OS	C3435T C1236T	8
Shim 2010	Korea	200 150/50 58 (19–76)	Gastric	TP (175 mg/m^2^ and 75 mg/m2 cisplatin)	RFLP	P >0.05	PFS/OS	C3435T C1236T	8
Szczyrek 2016	Poland	58 49/9 N/A	Lung	Docetaxel (75 mg/m^2^)	PCR HRM	P >0.05	OS	C3435T	8
Tian 2012	American	511 N/A 58 (24–87)	Ovary	TP (175 mg/m^2^ and AUC5-6) or TP (135 mg/m2 and 75 mg/m^2^ cisplatin)	Sequenom iPLEX Mass ARRAY Platform and MALDI-TOF Mass Spectrometry	P >0.05	PFS/OS	C3435T	9

## Discussion

This is the first meta-analysis to investigate the role of ABCB1 polymorphisms in patients administered taxane-containing chemotherapy. This comprehensive quantitative study included 15 studies involving 3,320 patients with a broad range of characteristics. The *rs1045642* and *rs1128503* polymorphisms are the most extensively studied SNPs in ABCB1. Our results indicated that C3435T polymorphisms did not affect PFS and OS, whereas subgroup analysis according to the geographical area, tumor type, and treatment regimen identified significant associations of the polymorphisms with PFS and OS. This finding is consistent with the results of two previous multicenter studies conducted in the United States and Japan. A meta-analysis reported by Jiang et al., (2018) revealed that the rs1045642 and rs1128503 polymorphisms were not related to the response to chemotherapy. Although heterogeneities were found in the overall analyses of PFS and OS in the C3435T homozygote and dominant models, subgroup analysis identified geographical area, tumor type, and chemotherapy regimen as the three main sources of heterogeneity between studies.

There are differences in the genetic and biological characteristics, clinical progression pattern, therapeutic responses, and prognoses among different tumor types; moreover, the distribution of paclitaxel varies among different tumors (Giordano et al., 2016). Sensitivity analysis revealed that the study by Li et al., (2017) was a source of statistical heterogeneity. When this outlier study was removed, there was no evidence of heterogeneity in the four remaining studies in the *C3435T* homozygote model. This observation may arise because Li et al., (2017) enrolled patients with breast cancer, which differs from the patient groups in the other studies. Therefore, it is important to analyze the relationship between *ABCB1 *and prognosis for different tumor types separately. Our study showed that in patients with ovarian cancer, being a *C3435T TT* carrier had a significant predictive value for the response to chemotherapy, as these patients had better OS and PFS. Wang et al., (2005) showed that the* rs1045642 *wild-type allele produces significantly higher *ABCB1 mRNA* levels, leading to higher P-gp expression levels; whereas patients with homozygous variants of C3435T exhibited reduced P-gp expression levels (Hoffmeyer et al., 2000). Penson et al., (2004) 1 reported that patients with ovarian cancer with high expression levels of P-gp had a shorter survival time and poorer prognosis than those with higher P-gp levels. *ABCB1* polymorphisms have different effects on the activities of different chemotherapeutic drugs, and the response to chemotherapy and prognosis following administration of different regimens vary widely. *ABCB1 *polymorphisms are related to the response to platinum-based chemotherapy (Sun et al., 2016; Zhang et al., 2018). Considering TP as a first-line chemotherapy regimen and platinum as a substrate of ABCB1, inconsistent effects may be observed when the chemotherapy regimen involves TP. Our study showed that TT carriers exhibited conflicting outcomes in patients treated with different regimens. Additionally, the level of medical care and medical resources in different regions are inconsistent, factors that will affect the prognosis of patients (Bray et al., 2018). 

Our study demonstrated the predictive value of *ABCB1 C1236T* for PFS and OS in patients who were administered taxane-containing chemotherapy. Our results were consistent with those reported by Bjorn et al., (2018) and Zhou et al., (2015). The effect of genetic variation on prognosis may be related to mutations that cause changes in the encoded amino acids, affecting the normal function of certain drug transporters, and leading to phenomena such as decreased expression of P-gp. These changes can lead to changes in the pharmacokinetics of a drug, and decreased levels of the drug pumped out of the cell, resulting in the accumulation of the drug in the cell, which may adversely affect therapeutic efficacy (2003). Bosch et al., (2006) reported that the *C1236T* homozygous mutant had decreased clearance of docetaxel, leading to an increase in the area under the curve. The C1236T polymorphism may also indirectly affect the stability of the mRNA (Frittitta et al., 2001). Shen et al., (1999) suggested that allele-specific differences in RNA folding influence downstream mRNA splicing, processing, or translational control and regulation, and reduced translational activity may also occur. 

There were some limitations to this study. The adjusted factors of the extracted data on survival time differed among the studies included, which may have affected the results for disease progression and death. Additionally, subgroup analyses based on other baseline characteristics of patients were not conducted, because these data were not available in the studies. The composite effects with other clinical factors and gene variants such as *G2677T/A *were not evaluated because of the unavailability of data. Despite these limitations, our meta-analysis showed that the precision of the estimation was improved by integrating multiple datasets and enlarging the sample size. Additionally, we found no publication bias, supporting the validity of the results of our meta-analysis. 

In conclusion, our study suggests a predictive role for *ABCB1* polymorphisms in the survival of patients undergoing taxane chemotherapy. Specifically, T gene carriers have a greater survival benefit. These results provide preliminary evidence of a correlation between the prognosis of patients with cancer and different geographical regions, tumor types, and chemotherapy regimens. Additional large-scale prospective studies are needed to evaluate the relationship between other factors such as specific populations, tumors, *G2677T/A* polymorphisms, and clinical cancer prognosis, to produce more accurate and robust conclusions.

## References

[B1] Auner V, Sehouli J, Oskay-Oezcelik G (2010). ABC transporter gene expression in benign and malignant ovarian tissue. Gynecol Oncol.

[B2] Bergmann TK, Green H, Brasch-Andersen C (2011). Retrospective study of the impact of pharmacogenetic variants on paclitaxel toxicity and survival in patients with ovarian cancer. Eur J Clin Pharmacol.

[B3] Bjorn N, Jakobsen Falk I (2018). ABCB1 variation affects myelosuppression, progression-free survival and overall survival in paclitaxel/carboplatin-treated ovarian cancer. Patients.

[B4] Bosch TM, Huitema AD, Doodeman VD (2006). Pharmacogenetic screening of CYP3A and ABCB1 in relation to population pharmacokinetics of docetaxel. Clin Cancer Res.

[B5] Bray F, Ferlay J, Soerjomataram I (2018). Global cancer statistics 2018: GLOBOCAN estimates of incidence and mortality worldwide for 36 cancers in 185 countries. CA Cancer J Clin.

[B6] Chang H, Rha SY, Jeung HC (2009). Association of the ABCB1 gene polymorphisms 2677G>T/A and 3435C>T with clinical outcomes of paclitaxel monotherapy in metastatic breast cancer patients. Ann Oncol.

[B7] Chang H, Rha SY, Jeung HC (2010). Association of the ABCB1 3435C>T polymorphism and treatment outcomes in advanced gastric cancer patients treated with paclitaxel-based chemotherapy. Oncol Rep.

[B8] De Iudicibus S, De Pellegrin A, Stocco G (2008). ABCB1 gene polymorphisms and expression of P-glycoprotein and long-term prognosis in colorectal cancer. Anticancer Res.

[B9] Dean M, Rzhetsky A, Allikmets R (2001). The human ATP-binding cassette (ABC) transporter superfamily. Genome Res.

[B10] Egger M, Davey Smith G, Schneider M (1997). Bias in meta-analysis detected by a simple, graphical test. BMJ.

[B11] Frittitta L, Ercolino T, Bozzali M (2001). A cluster of three single nucleotide polymorphisms in the 3’-untranslated region of human glycoprotein PC-1 gene stabilizes PC-1 mRNA and is associated with increased PC-1 protein content and insulin resistance-related abnormalities. Diabetes.

[B12] Gandara DR, Kawaguchi T, Crowley J (2009). Japanese-US common-arm analysis of paclitaxel plus carboplatin in advanced non-small-cell lung cancer: a model for assessing population-related pharmacogenomics. J Clin Oncol.

[B13] Genovese I, Ilari A, Assaraf YG (2017). Not only P-glycoprotein: Amplification of the ABCB1-containing chromosome region 7q21 confers multidrug resistance upon cancer cells by coordinated overexpression of an assortment of resistance-related proteins. Drug Resist Updat.

[B14] Giordano S, Zucchetti M, Decio A (2016). Heterogeneity of paclitaxel distribution in different tumor models assessed by MALDI mass spectrometry imaging. Sci Rep.

[B15] Grau JJ, Caballero M, Campayo M (2009). Gene single nucleotide polymorphism accumulation improves survival in advanced head and neck cancer patients treated with weekly paclitaxel. Laryngoscope.

[B16] Higgins JP, Thompson SG, Deeks JJ (2003). Measuring inconsistency in meta-analyses. BMJ.

[B17] Hitzl M, Drescher S, van der Kuip H (2001). The C3435T mutation in the human MDR1 gene is associated with altered efflux of the P-glycoprotein substrate rhodamine 123 from CD56+ natural killer cells. Pharmacogenetics.

[B18] Hoffmeyer S, Burk O, von Richter O (2000). Functional polymorphisms of the human multidrug-resistance gene: multiple sequence variations and correlation of one allele with P-glycoprotein expression and activity in vivo. Proc Natl Acad Sci U S A.

[B19] Jiang Q, Xu M, Liu Y (2018). Influence of the ABCB1 polymorphisms on the response to Taxane-containing chemotherapy: a systematic review and meta-analysis. Cancer Chemother Pharmacol.

[B20] Johnatty SE, Beesley J, Gao B (2013). ABCB1 (MDR1) polymorphisms and ovarian cancer progression and survival: A comprehensive analysis from the Ovarian Cancer Association Consortium and the Cancer Genome Atlas. Gynecol Oncol.

[B21] Johnatty SE, Beesley J, Paul J (2008). ABCB1 (MDR 1) polymorphisms and progression-free survival among women with ovarian cancer following paclitaxel/carboplatin chemotherapy. Clin Cancer Res.

[B22] Karageorgopoulou S, Kostakis ID, Gazouli M (2017). Prognostic and predictive factors in patients with metastatic or recurrent cervical cancer treated with platinum-based chemotherapy. BMC Cancer.

[B23] Kelland L (2007). The resurgence of platinum-based cancer chemotherapy. Nat Rev Cancer.

[B24] Kim JW, Kim JH, Im SA (2012). ABCB1, FCGR2A, and FCGR3A polymorphisms in patients with HER2-positive metastatic breast cancer who were treated with first-line taxane plus trastuzumab chemotherapy. Oncology (Switzerland).

[B25] Kim RB, Leake BF, Choo EF (2001). Identification of functionally variant MDR1 alleles among European Americans and African Americans. Clin Pharmacol Ther.

[B26] Leschziner GD, Andrew T, Pirmohamed M (2007). ABCB1 genotype and PGP expression, function and therapeutic drug response: a critical review and recommendations for future research. Pharmacogenomics J.

[B27] Li W, Zhang D, Du F (2017). ABCB1 3435TT and ABCG2 421CC genotypes were significantly associated with longer progression-free survival in Chinese breast cancer patients. Oncotarget.

[B28] Liu KJ, He JH, Su XD (2013). Saracatinib (AZD0530) is a potent modulator of ABCB1-mediated multidrug resistance in vitro and in vivo. Int J Cancer.

[B29] Mizuno N, Niwa T, Yotsumoto Y (2003). Impact of drug transporter studies on drug discovery and development. Pharmacol Rev.

[B30] Moher D, Liberati A, Tetzlaff J (2009). Preferred reporting items for systematic reviews and meta-analyses: the PRISMA statement. PLoS Med.

[B31] Orina JN, Calcagno AM, Wu CP (2009). Evaluation of current methods used to analyze the expression profiles of ATP-binding cassette transporters yields an improved drug-discovery database. Mol Cancer Ther.

[B32] Peethambaram P, Fridley BL, Vierkant RA (2011). Polymorphisms in ABCB1 and ERCC2 associated with ovarian cancer outcome. Int J Mol Epidemiol Genet.

[B33] Penson RT, Oliva E, Skates SJ (2004). Expression of multidrug resistance-1 protein inversely correlates with paclitaxel response and survival in ovarian cancer patients: a study in serial samples. Gynecol Oncol.

[B34] Priyadarshini R, Raj GM, Kayal S (2019). Influence of ABCB1 C3435T and C1236T gene polymorphisms on tumour response to docetaxel-based neo-adjuvant chemotherapy in locally advanced breast cancer patients of South India. J Clin Pharm Ther.

[B35] Qiao R, Wu W, Lu D (2016). Influence of single nucleotide polymorphisms in ABCB1, ABCG2 and ABCC2 on clinical outcomes to paclitaxel-platinum chemotherapy in patients with non-small-cell lung cancer. Int J Clin Exp Med.

[B36] Schaich M, Kestel L, Pfirrmann M (2009). A MDR1 (ABCB1) gene single nucleotide polymorphism predicts outcome of temozolomide treatment in glioblastoma patients. Ann Oncol.

[B37] Shen LX, Basilion JP, Stanton VP Jr. (1999). Single-nucleotide polymorphisms can cause different structural folds of mRNA. Proc Natl Acad Sci U S A.

[B38] Shim HJ, Yun JY, Hwang JE (2010). BRCA1 and XRCC1 polymorphisms associated with survival in advanced gastric cancer treated with taxane and cisplatin. Cancer Sci.

[B39] Sun S, Cai J, Yang Q (2016). Prognostic value and implication for chemotherapy treatment of ABCB1 in epithelial ovarian cancer: A Meta-Analysis. PLoS One.

[B40] Szczyrek M, Mlak R, Krawczyk P (2017). Polymorphisms of genes encoding multidrug resistance proteins as a predictive factor for second-line docetaxel therapy in advanced non-small cell lung cancer. Pathol Oncol Res.

[B41] Tian C, Ambrosone CB, Darcy KM (2012). Common variants in ABCB1, ABCC2 and ABCG2 genes and clinical outcomes among women with advanced stage ovarian cancer treated with platinum and taxane-based chemotherapy: A Gynecologic Oncology Group study. Gynecol Oncol.

[B42] Tierney JF, Stewart LA, Ghersi D (2007). Practical methods for incorporating summary time-to-event data into meta-analysis. Trials.

[B43] Wang D, Johnson AD, Papp AC (2005). Multidrug resistance polypeptide 1 (MDR1, ABCB1) variant 3435C>T affects mRNA stability. Pharmacogenet Genomics.

[B44] Xia YZ, Yang L, Xue GM (2016). Combining GRP78 suppression and MK2206-induced Akt inhibition decreases doxorubicin-induced P-glycoprotein expression and mitigates chemoresistance in human osteosarcoma. Oncotarget.

[B45] Zhang Z, Xiang Q, Mu G (2018). XRCC1 polymorphism and overall survival in ovarian cancer patients treated with platinum-based chemotherapy: A systematic review and MOOSE-compliant meta-analysis. Medicine (Baltimore).

[B46] Zhong J, Guo Z, Fan L (2019). ABCB1 polymorphism predicts the toxicity and clinical outcome of lung cancer patients with taxane-based chemotherapy. Thorac Cancer.

[B47] Zhou Z, Chen Q, Zuo D (2015). ABCB1 (rs1128503) polymorphism and response to chemotherapy in patients with malignant tumors-evidences from a meta-analysis. Int J Clin Exp Med.

